# Exosomal transfer of miR-15b-3p enhances tumorigenesis and malignant transformation through the DYNLT1/Caspase-3/Caspase-9 signaling pathway in gastric cancer

**DOI:** 10.1186/s13046-019-1511-6

**Published:** 2020-02-10

**Authors:** Shuchun Wei, Lei Peng, Jiajia Yang, Huaiming Sang, Duochen Jin, Xuan Li, Meihong Chen, Weifeng Zhang, Yini Dang, Guoxin Zhang

**Affiliations:** Department of Gastroenterology, The First Affiliated Hospital of Nanjing Medical University, Nanjing, 210029 China

**Keywords:** Exosomes, miR-15b-3p, Gastric cancer, DYNLT1, Apoptosis, Cleaved Caspase-3, Cleaved Caspase-9

## Abstract

**Background:**

Exosomes are essential for tumor growth, metastasis, and are used as novel signaling molecules in targeted therapies. Therefore, exosomal miRNAs can be used in new diagnostic and therapeutic approaches due to their involvement in the development of cancers. However, the detailed biological function, potential molecular mechanism and clinical application of exo-miR-15b-3p in gastric cancer (GC) remains unclear.

**Methods:**

miR-15b-3p mRNA levels in tissues, serum, cells and exosomes were analyzed using qRT-PCR assays. qRT-PCR, immunohistochemical and western blotting analyses were utilized for the determination of DYNLT1 expression. The interrelationship connecting miR-15b-3p with DYNLT1 was verified using Dual-luciferase report, western blotting and qRT-PCR assays. Fluorescent PKH-26 or GFP-Lv-CD63 labeled exosomes, as well as Cy3-miR-15b-3p, were utilized to determine the efficacy of the transfer of exo-miR-15b-3p between BGC-823 and recipient cells. Several in vitro assays and xenograft tumor models were conducted to determine exo-miR-15b-3p impact on GC progression.

**Results:**

This is the first study to confirm high miR-15b-3p expression in GC cell lines, tissues and serum. Exosomes obtained from 108 GC patient serum samples and GC cell-conditioned medium were found to show upregulation of exo-miR-15b-3p, with the area under the ROC curve (AUC) being 0.820 [0.763–0.876], which is superior to the AUC of tissues and serum miR-15b-3p (0.674 [0.600–0.748] and 0.642 [0.499–0.786], respectively). In addition, high exo-miR-15b-3p expression in serum was found to accurately predict worse overall survival. SGC-7901 and GES-1 cells are capable of internalizing BGC-823 cell-derived exosomes, allowing the transfer of miR-15b-3p. Migration, invasion, proliferation and inhibition of apoptosis in vitro and in vivo were enhanced by exo-miR-15b-3p, by restraining DYNLT1, Cleaved Caspase-9 and Caspase-3 expression.

**Conclusions:**

This study identified a previously unknown regulatory pathway, exo-miR-15b-3p/DYNLT1/Caspase-3/Caspase-9, which promotes GC development and GES-1 cell malignant transformation. Therefore, serum exo-miR-15b-3p may be a potential GC diagnosis and prognosis biomarker, which can be used in precise targeted GC therapy.

## Introduction

Worldwide, gastric cancer (GC) frequency is fourth highest among malignancies and the second most-likely cause of cancer-related death [1], as well as the second most frequent cancer in China [2]. Although, GC diagnosis, as well as treatment methods have improved greatly during recent times, the GC patient five-year survival rate is reported to be 10–30%, due to diagnosis delays [1, 3]. GC development and progression are regulated by a variety of factors, such as genetics, epigenetics and the environment [4, 5]. Due to its high complexity, current treatment methods, including surgery, chemotherapy, and radiotherapy, are not yet able to achieve satisfactory therapeutic outcomes [6]. Therefore, identifying sensitive and specific biomarkers for GC diagnosis and identifying GC progression related molecular mechanisms are critical for early diagnosis and effective targeted therapy of GC.

As small non-coding RNAs, microRNAs (miRNAs) can function as vital posttranscriptional mRNA translation and gene expression regulators in most cell types [7]. miRNAs are found in serum and other body fluids, and function as biomarkers of diseases due to their differential expression between patients and healthy individuals [8]. Exosomes are extracellular vesicles with an average diameter of 30–200 nm that have the same topology as the cell and contain a specific composition of proteins, lipids, nucleic acids and glycoconjugates [9]. They are derived from endocytic membranes and serve as vehicles for cell-to-cell communication, remodeling the extracellular environment or transmitting signals and molecules to neighboring recipient cells [9, 10]. Due to their potential use in numerous pathological and physiological processes of various diseases, differences in exosome function between healthy and diseased individuals has attracted much attention from researchers [9–11]. Interestingly, exosomes can carry numerous miRNAs that act locally or enter into circulation to act at distal sites, since internal miRNAs are protected from being digested by RNase, as a result of the protection offered by the lipid membrane of the exosomes [12, 13]. New evidence has demonstrated that exosomal miRNAs (exo-miRNAs) transmitted between cells perform a crucial regulatory function in apoptosis, invasion, migration, proliferation, as well as chemoresistance of multifarious tumors, including GC [13–17].

The correlation between miR-15b-3p and GC development has not been demonstrated in any previous study. In this current study, exosomal miR-15b-3p (exo-miR-15b-3p) was found to be released by BGC-823 cells, promoting GC progression and the malignant transformation of GES-1 (normal gastric mucosa epithelium cells), by regulating the DYNLT1/Caspase-3/Caspase-9 axis. Additionally, the potential use of serum exo-miR-15b-3p for the diagnosis and prognosis of GC in the form of a liquid biological marker was also demonstrated. Thus, this study provides a novel target and perspective for GC diagnosis and prognosis through effective targeted therapies.

## Materials and methods

### Specimens of a clinical nature

Histologically confirmed GC tissue and paired adjacent noncancerous tissue were obtained from 108 patients undergoing surgical procedures at Nanjing Medical University’s First Affiliated Hospital in China. The 108 patients mentioned above, were gender, age and disease history matched with 108 non-GC volunteers, who provided human serum samples. All clinical specimens were collected under the guidance of the Health Insurance Portability and Accountability Act (HIPAA) protocol and were stored at − 80 °C after being frozen in liquid nitrogen, once collected. First Affiliated Hospital of Nanjing Medical University Ethics Committee Approval was obtained to conduct this study, while written consent was obtained from all participants.

### Cell culture

The following three GC cell lines: normal GES-1 gastric mucosa epithelium cell line; moderately differentiated adenocarcinoma SGC-7901 cell line and the poorly differentiated adenocarcinoma BGC-823 cell line, were purchased from the Cell Bank of Type Culture Collection of the Chinese Academy of Sciences. The cells were cultured at 37 °C, in RPMI 1640 medium supplemented with 1% penicillin/streptomycin, 10% fetal bovine serum (FBS) and 5% CO_2_. All culture medium reagents were obtained from Gibco, USA.

### Isolation and characterization of exosomes

After the cells had reached a confluency of 70–80%, the medium was changed to a RPMI 1640 medium with 10% exosome-depleted FBS (obtained through ultracentrifugation at 120,000×g at 4 °C for 6 h [18]). After 48 h, 50 ml of the conditioned medium (CM) was collected from each cell line, and ultracentrifugation was used to extract exosomes from the medium, following previously described standard procedures [19]. In order to collect blood samples for serum exosome isolation, ethylenediaminetetraacetic acid (EDTA) containing collection tubes were used. Within an hour, the tubes were centrifuged at 1900×g at 4 °C for 10 min, using a swinging bucket rotor. A new tube was used to collect the upper (yellow) serum phase, and 16,000×g centrifugation at 4 °C for 10 min was conducted to eliminate additional cellular fragments, as well as cell debris. Then, an exoEasy Maxi Kit (Qiagen, Hilden, Germany; Cat. Number: 76064) was used, as instructed by the manufacturer, to isolate serum exosomes. As described in a previous study [20], a FEI Tecnai T20 transmission electron microscope (TEM) (FEI Company, USA) was used to observe the exosomes, while a Nano Sight NS 300 system (Nano Sight Technology, Malvern, UK) was used to determine exosome quantity and size.

### Extraction of RNA and quantitative reverse transcription (qRT)-PCR assays

TRIzol reagent (Invitrogen, USA) was used to extract total RNA from tissues, cells and CM derived-exosomes, which were purified using a miRNeasy Serum/Plasma Kit (Qiagen, Germany; Cat. Number: 217184), as instructed by the manufacturer. In addition, exosomal RNA was isolated directly from serum, using an exoRNeasy Serum/Plasma MidiKit (Qiagen, Hilden, Germany; Cat. Number: 77044). The miRNeasy Serum/Plasma Spike-In Control (cel-miR-39, Qiagen, Hilden, Germany; Cat. Number: 219610) was used as the serum miRNA expression profiling internal control, as instructed by the manufacturer. The cDNA of the RNAs were created with the aid of a PrimeScript™ RT Reagent Kit (TaKaRa, Japan; Code No. RR037A (miRNAs)/RR036A (mRNAs)). TB Green® Premix Ex Taq™ (TaKaRa, Japan, Code No. RR420A) was used to conduct the qRT -PCR, with the results recorded using ABI StepOne™ Software v2.3 (Applied Biosystems, USA). GAPDH functioned as an internal control for DYNLT1 mRNA levels and the relative miR-15b-3p expression of serum-exosomes were normalized to cel-miR-39, which was normalized to U6 in CM-exosomes, cells and tissues. The 2^−ΔCT^ formula was used to determine gene expression fold change. Additional file 7: Table S1 lists all primary sequences used.

### Oligonucleotide transfection

Lipofectamine2000 Reagent (Invitrogen, USA) and Opti-MEM (Gibco, USA) were used, as instructed by the manufacturer, in 6-well plates to transfect the GenePharma Corporation (SGC, China) synthesized miR-15b-3p mimics/scrambled negative control RNA (NC) or miR-15b-3p inhibitor/scrambled negative control RNA (inhibitor-NC) into cells. After 48 h and 24 h of oligonucleotide transfection, cells were harvested to isolate total cell lysates and total RNA for western blotting and qRT-PCR analyses, in order to determine DYNLT1 and miR-15b-3p levels, respectively. The miR15b-3p mimics and inhibitor sequences mentioned above are listed in Additional file 7: Table S2.

### Lentivirus infection

Genechem Inc. (China) constructed luciferase-labelled lentivirus vectors carrying miR-15b-3p (Lv-miR-15b-3p)/negative control (Lv-NC), miR-15b-3p inhibitor (Lv-inhibitor)/negative control (Lv-inNC) and GFP-labelled lentivirus vectors containing CD63 (GFP-Lv-CD63) were used. BGC-823 cells were infected in 6-well plates, using 10 μl of the aforementioned lentiviral vectors for 3 days at 37 °C. Then, selection of successful lentiviral transfected cells was done using 1.0 μg/ml puromycin (Sigma Aldrich, USA). The primers used for the amplification of miR-15b-3p were: 5′-.

AGGTATGCACGCGTGAATTGTTACTTTTTTTTCTATAAAGCTAGGTTGG - 3′ (sense) and 5′-GCCGACACGGGTTAGGATCAAAAAACACTACGCCAATATTTA-CGTGC-3′(antisense). Sequences used for the Lv-miR-15b-3p inhibitor were: 5′-AATTCAAAAACGAATCATTATTTGCTGCTCTA-3′ (sense) and 5′-CCGGTAGAGCAGCAAATAATGATTCGTTTTTG-3′(antisense). qRT-PCR was performed to validate infection efficiency.

### Proliferation assay

Into 6-well plates, the harvested cells were added at a concentration of 1 × 10^3^ cells/well, for 10–15 days, to be used for the colony formation assay. Fixation of the colonies were done using 2 ml of paraformaldehyde for 30 min, while 0.1% crystal violet was used for 30 min at room temperature for cell staining. In addition, a Cell-Light EdU Apollo567 In Vitro Kit (RiboBio, China) and a Cell Counting Kit-8 (CCK-8) kit (Dojindo Laboratories, Japan) were utilized to evaluate the proliferation of the cells. For the CCK-8 assay, into each well of a 96-well plate containing 2 × 10^3^ transfected cells, 10 μL of CCK-8 reagent was added at the same time every day for further incubation (2 h). A Microplate reader (ELX-800; Bio-Tek, USA) was used to measure absorption at 450 nm, at a series of time points (0, 24, 36, 48, 72 and 96 h). For the 5-ethynyl-2′-deoxyuridine (EdU) assay, rigorous processing was carried out on the cells in 96-well plates with the cells at a concentration of 2 × 10^4^ cells/well, as instructed by the manufacturer [21]. Finally, a Nikon ECLIPSE E800 fluorescence microscope was used to examine the cell samples.

### Apoptosis assay

An Annexin V-PI apoptosis detection kit (Vazyme Biotech Co. Ltd., China) was used in a manner similar to that of a previous description [22, 23] to detect apoptosis. Thereafter, fluorescence-activated cell sorting (FACS) was utilized to count the stained cells using CellQuest software (BD Biosciences, USA) connected to a Calibur flow cytometer. A TUNEL FITC Apoptosis Detection Kit (Vazyme Biotech Co. Ltd., China) was used, as instructed by the manufacturer, to conduct TUNEL staining. Immunofluorescence was observed using a Nikon ECLIPSE E800 fluorescence microscope.

### Transwell assay

First, into a 24-well plate, transwell assay inserts (Millipore, USA) were added. A Matrigel-coated membrane (50 μL/well, BD Biosciences, Franklin Lakes, NJ) was used for the invasion assay, while a normal membrane was used for the migration assay, as the apical chamber membrane. Then, 600 μL of 10% FBS containing medium was seeded into the basolateral chamber, and 100 μL of FBS-free RPMI 1640 medium (Gibco, USA) was added into the apical chamber containing 2 × 10^5^ cells in each well to re-suspend the cells. After incubation for 24 h at 37 °C, PBS was used to rinse the Transwell plates twice, fix with 4% paraformaldehyde for 30 min, while 0.1% crystal was used for 30 min at room temperature, for staining. Subsequently, utilizing an inverted light microscope the cells were observed, photographed and counted.

### Luciferase reporter assay

The pmirGLO dual-luciferase miRNA target expression vector (Promega, USA) was transfected with the PCR amplified 3′ untranslated regions (3′-UTR) of DYNLT1 mRNA. In 24-well plates, the luciferase construct containing wild-type (WT) or mutated binding site of DYNLT1 (constructed by Genechem Inc., China) were transfected into target cells. This was followed by co-transfection with miR-15b-3p mimics, inhibitor, NC or inhibitor-NC using Lipofectamine2000, to identify the binding site between DYNLT1 and miR-15b-3p. Determination of luciferase activity after 48 h of transfection and normalization with Renilla luciferase was done utilizing a Dual-Luciferase Reporter System Kit (E1910, Promega, USA), as previously reported [24].

### Western blotting analysis

Protein extraction from cells, tissues, and exosomes were performed using a radioimmunoprecipitation assay (RIPA) kit (Sigma-Aldrich, USA), as instructed by the manufacturer. After determination of protein concentration using a bicinchoninic acid (BCA) kit (Pierce, USA), SDS-containing polyacrylamide gel (SDS-PAGE) was used for the separation of equal amounts (35 μg for cells and tissues, and 10 μg for exosome pellets) of protein samples. Thereafter, the samples were moved onto polyvinylidene difluoride (PVDF) membranes (Bio-Rad, USA). Then, for 1 h, 5% non-fat milk in TBSTween (TBST) (0.1 M, pH 7.4) was used to block the membranes, followed by hybridization with primary antibodies against CD9 (ab92726, 1:1000 dilution), CD63 (ab217345, 1:1000 dilution), DYNLT1 (ab202583, 1:2000 dilution), BAX (ab32503, 1:1000 dilution), BCL-2 (ab32124, 1:1000 dilution) and TSG101 (ab125011, 1:1000 dilution), from Abcam (USA); Cleaved caspase-3 (9664, 1:1000 dilution) and Cleaved caspase-9 (7237, 1:1000 dilution), from Cell Signaling Technology (USA), overnight at 4 °C. The antibodies for GAPDH (QYA03819B, 1:2000 dilution) and β-Actin (sc-47,778, 1:1000 dilution) from Santa Cruz Biotechnology (USA) served as reference proteins. The immunocomplexes were incubated with corresponding horseradish peroxidase conjugated secondary antibodies (Applygen, China; 1:2000 dilution), for 2 h at room temperature. Thereafter, an enhanced chemiluminescence assay was conducted on a SuperSignal™ West Femto Maximum Sensitivity Substrate (34,095, Thermo Fisher, USA) to visualize the blots.

### Exosome labeling and uptake

The cells cultured on four-well chamber slides were washed with PBS thrice, fixed using 4% paraformaldehyde for 15 min, once again washed with PBS, and permeabilized using 0.5% Triton-X 100 (dissolved in PBS) for 20 min. For exosome tracking, exosomes secreted by the BGC-823 cells were labeled using PKH26 red fluorescent dye (Sigma-Aldrich, USA) or exosomal marker, CD63 (green; Genechem Inc., China), while F-actin was stained using phalloidin-FITC (green), and DAPI (blue) was used to label nuclei. Cy3-(miR-15b-3p inhibitor/inhibitor-NC/mimics/NC) were synthesized, as well as purified by RiboBio Co. (China). A Nikon ECLIPSE E800 fluorescence microscope was used to capture images. The uptake capacity of SGC-7901 and GES-1 into exosomes containing different miRNA sequences (mimics/NC/inhibitor/inhibitor-NC) was determined using immunofluorescence assays and qRT-PCR.

### Animal studies

6–8 week old BALB/c-nu male nude mice were kept in an animal facility that was pathogen-free and were randomly divided into five groups (*n* = 5). The groups received subcutaneous injections of miR-15b-3p enriched/Lv-NC exosomes, miR-15b-3p inhibited/Lv-inNC exosomes (1 × 10^9^ exosomes/ml) or PBS treated SGC-7901 cells (2 × 10^6^ cells in 200 μl PBS). The anesthetization of the mice was done using xylazine (10 mg/kg) or ketamine (100 mg/kg), while bioluminescence signals were observed using an IVIS 100 Imaging System (Xenogen, USA) 15 min after D-luciferin (100 mg/kg, Xenogen, USA) was injected into the mice. Once in 4 days, a digital caliper was used to measure the tumors and the following formula was used to calculate tumor volume: (width^2^ × length)/2, until euthanasia, 28 days after cell inoculation. Finally, the subcutaneous tumors of the mice were excised and at room temperature were frozen in liquid nitrogen or fixed in 4% paraformaldehyde for subsequent studies. Approved protocols provided by Nanjing Medical University’s Institutional Animal Care and Research Advisory Committee were followed for all animal experiments.

### Immunohistochemistry

The tumor masses of both mice and clinical samples were 4% paraformaldehyde fixed, paraffin embedded at 58 °C and cut into 4 μm sections, followed by staining with anti-DYNLT1 antibodies (1:50 dilution, ab202583, Abcam, USA). Aperio Scan-Scope AT Turbo (Aperio, USA) was used to capture images of the tumors, while image-scope software (Media Cybernetics Inc.) was used to conduct the quantitative analysis.

### Statistical analysis

GraphPad Prism 7.00 Software (USA) and SPSS version 22.0 (SPSS, USA) were used to conduct the statistical analyses. Expression is presented as mean ± SEM of at least three independent experiments for all results. One-way analysis of variance (ANOVA) or student’s t test was performed to determine statistical differences among two or more groups. Sensitivity, specificity, and area under the curve (AUC), including 95% confidence interval (CI), were computed with the aid of the constructed receiver-operating characteristic (ROC) curves, using the Youden index (J) [25] to calculate the optimum cut-off values. The survival analysis included log-rank tests and Kaplan–Meier analyses. A *P* value of < 0.05 was used to indicate a statistically significant result. For all figures: *, *P* < 0.05; **, *P* < 0.01; &**, *P* < 0.001; and &&**, *P* < 0.0001.

## Results

### MiR-15b-3p is upregulated in GC

**Fig. 1:**
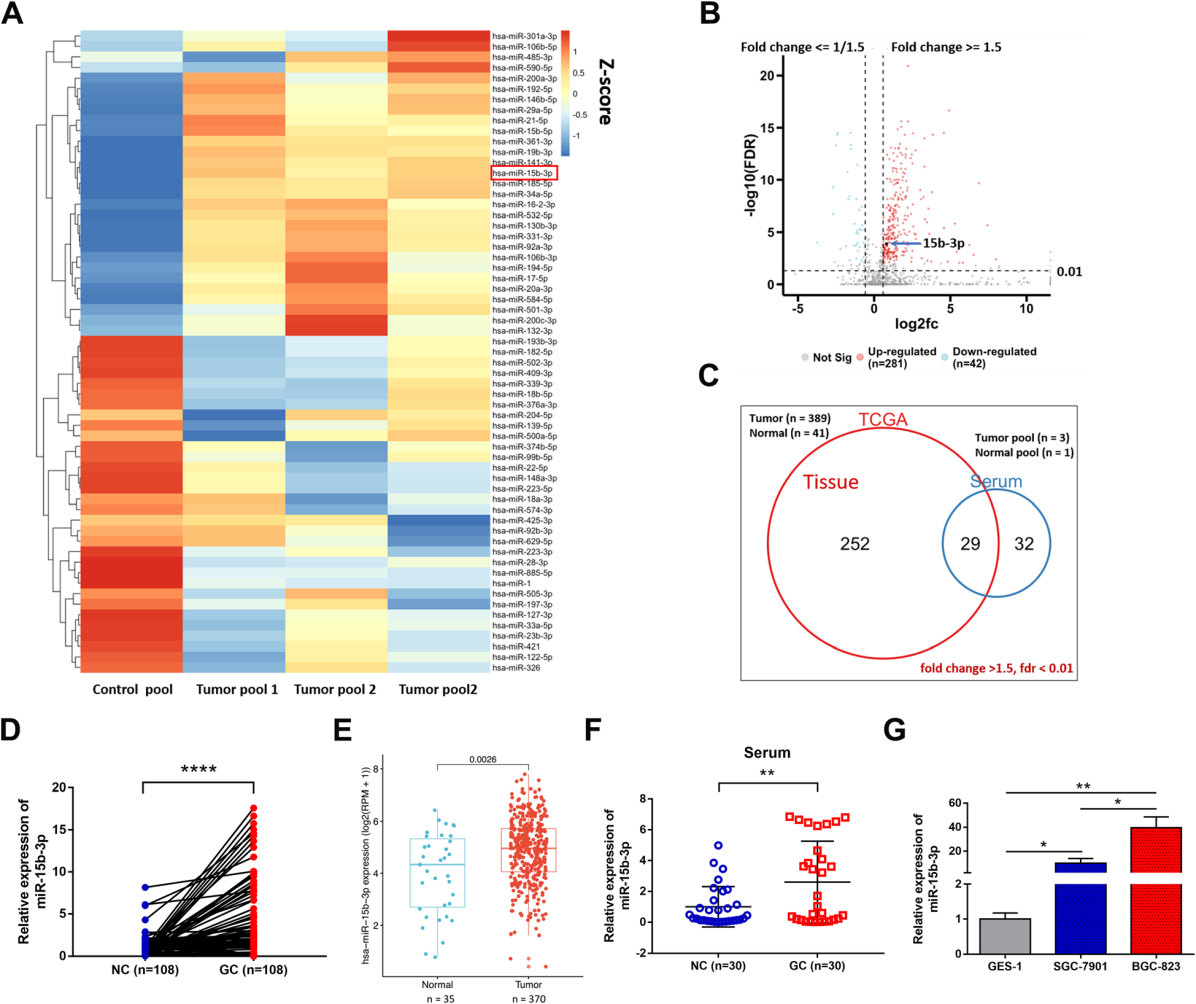
Increased expression of miR-15b-3p in GC. **a.** Heat map showing the miRNA expression profile, and the top 61 significantly upregulated miRNAs in the GC samples are listed. **b.** The volcano plot shows the difference between miRNAs in the tissues of healthy individuals and GC patients in the TCGA database. The miRNAs were classified based on fold change (log2fc) between the two groups. **c.** Venn diagram shows that the 29 miRNAs are significant highly expressed in both GC tissue (according to TCGA database) and microarray sequencing results. **d.** qRT-PCR analysis of has-miR-15b-3p expression by in 108 GC tissues and paired adjacent non-GC tissues. **e.** has-miR-15b-3p expression was found to be statistically significant in GC tissues of the TCGA database. **f.** GC patient serum and healthy volunteer (*n* = 30) serum miR-15b-3p levels were analyzed using qRT-PCR. **g.** qRT-PCR was used to detect miR-15b-3p expression in BGC-823, SGC-7901 and GES-1 cells. Mean ± SEM of the results are presented

Gene Expression Omnibus (GEO) database microarray data (accession number: GSE86226) were analyzed and the top 61 highly expressed miRNAs (fold change > 1.5, FDR < 0.01), in comparison with 3 pooled peripheral serum samples from 30 GC patients and 1 pooled sample from 10 controls (Fig. 1a). 281 miRNAs with highly significant expression were identified after the same criteria (fold change > 1.5, FDR < 0.01) for GC tissues was applied to the TCGA database (Fig. 1b). 29 miRNAs were found to fall into the intersection between the two sets of data (Fig. 1c). Among them, miR-15b-3p was the most prominent in the tumor tissues of 108 GC patients (Additional file 1: Figure S1a-l and Fig. 1d), which is consistent with the expression trend in the TCGA database (Fig. 1e). Table 1 shows the baseline characteristics of the 108 GC patients. Next, significantly higher miR-15b-3p levels were found in GC serum, compared with that of normal serum using qRT-PCR assay (*n* = 30, Fig. 1f). In addition, compared with the GES-1 cell line (Fig. 1g), distinctively elevated expression of miR-15b-3p was found in the moderately differentiated adenocarcinoma SGC-7901 cell line and the poorly differentiated adenocarcinoma BGC-823 cell line. Considering the significantly higher miR-15b-3p expression in BGC-823 cells, above that of SGC-7901 cells, we hypothesized that the miR-15b-3p expression is higher in GC cell lines with high malignancy. Collectively, these results show that GC development may involve miR-15b-3p. Hence, we focused on the functional role of miR-15b-3p.

**Table 1:** Clinicopathological features of 108 non-GC and 108 GC patients

Variables	NC = 108	GC = 108	*P* value
Age, years	60.64 ± 1.43	62.54 ± 0.91	0.260
Gender			1.000
Male	71(65.7%)	71(65.7%)	
Female	37(34.3%)	37(34.3%)	
Smoking			0.002*
Yes	17(15.7%)	37(34.3%)	
No	91(84.3%)	71(65.7%)	
Alcohol abuse			0.012*
Yes	12(11.1%)	26(24.1%)	
No	96(88.9%)	82(75.9%)	
Family history of cancer			0.000*
Yes	2(1.9%)	19(17.6%)	
No	106(98.1%)	89(82.4%)	
Hypertension			0.317
Yes	41(38.0%)	34(31.5%)	
No	67(62.0%)	74(68.5%)	
Diabetes mellitus			0.621
Yes	25(23.1%)	22(20.4%)	
No	83(76.9%)	86(79.6%)	
Heart disease			1.181
Yes	10(9.3%)	5(4.6%)	
No	98(90.7%)	103(95.4%)	
Pulmonary disease			0.269
Yes	5(4.6%)	9(8.3%)	
No	103(95.4%)	99(91.7%)	
History of taking NSAIDs			0.249
Yes	2(1.9%)	5(4.6%)	
No	106(98.1%)	103(95.4%)	

*P <0.05

### MiR-15b-3p overexpression enhances GC cell proliferation, invasion, migration and inhibits apoptosis

**Fig. 2:**
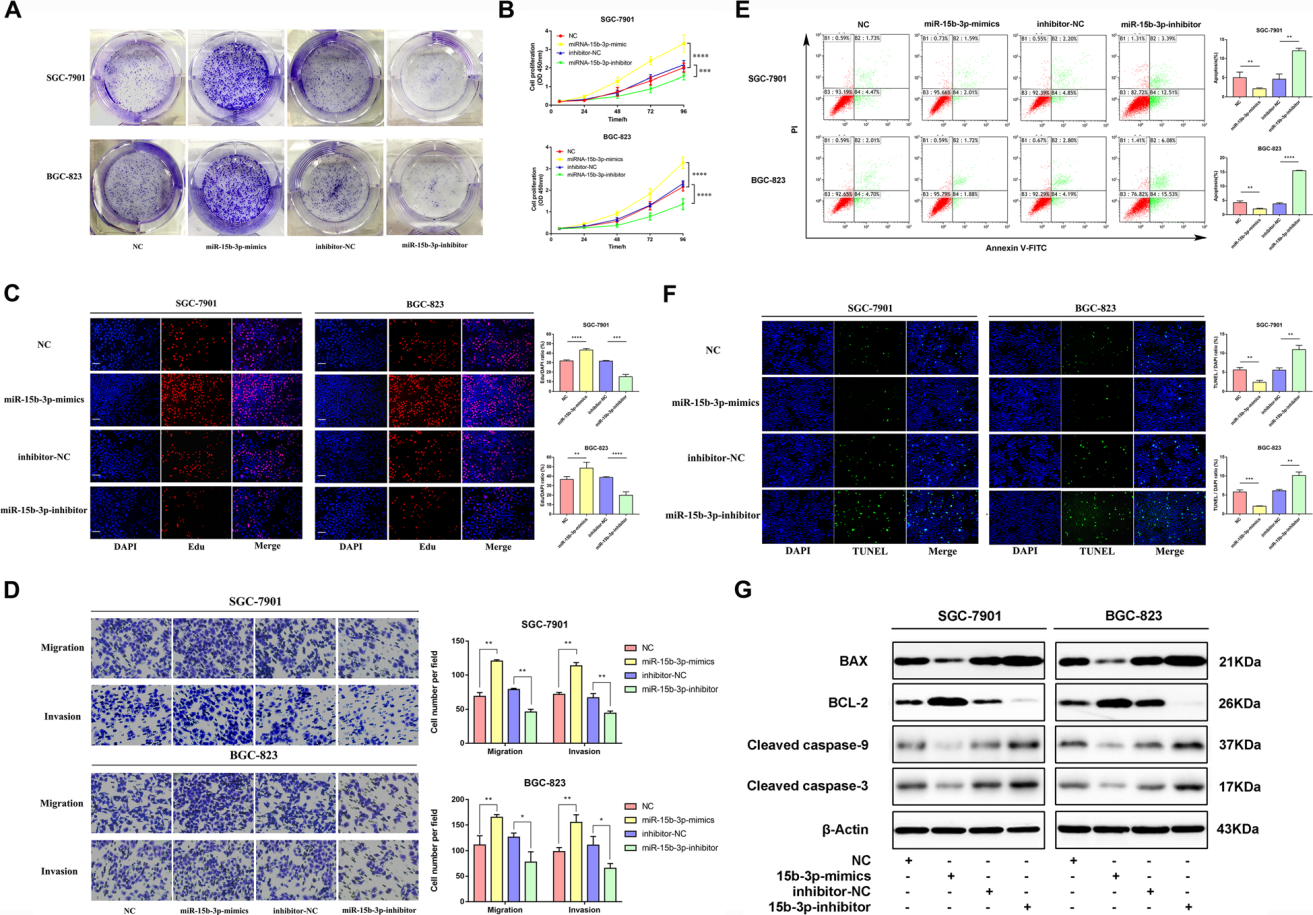
Upregulated miR-15b-3p levels enhance GC cell migration, invasion and proliferation, while inhibiting their apoptosis. miR-15b-3p action on SGC-7901 and BGC-823 cell proliferation was measured using colony formation (**a**), CCK-8 (**b**) and EdU (**c**) assays. Scale bar, 100 μm. **d** Transwell assays of miR-15b-3p mimics/NC/inhibitor/inhibitor-NC transfected BGC-823 and SGC-7901 cells. The cells that had migrated and had been invaded were counted and representative images are shown. **e** The BGC-823 and SGC-7901 cells were stained after 24 h of miR-15b-3p inhibitor/inhibitor-NC/mimics/NC treatment. Flow cytometry was used for analysis. The early apoptotic cell ratio (%) was recorded and is presented in the column chart. **f** TUNEL analysis was also used to measure BGC-823 and SGC-7901 cell apoptosis, when subjected to different treatments. Scale bar, 100 μm. **g** miR-15b-3p mimics/NC/inhibitor/inhibitor-NC transfected BGC-823 and SGC-7901 cells were subjected to western blotting analysis to detect apoptosis-related proteins, BAX, BCL-2, Cleaved caspase-9 and Cleaved caspase-3 levels. β-Actin was utilized as the loading control. Mean ± SEM of three independent experiments are presented

In order to determine whether miR-15b-3p plays a role in GC progression, we first investigated its effect on GC cell proliferation. Additional file 2 Figure S2 shows miR-15b-3p expression after transfection into SGC-7901 and BGC-823 cells. Results of the colony formation, CCK-8 and 5-ethynyl-2′-deoxy-uridine (EdU) assays reveal that in comparison with the respective control groups, treatment with miR-15b-3p mimics accelerate the proliferation of SGC-7901 and BGC-823 cells, while the miR-15b-3p inhibitor significantly inhibits their proliferation (Fig. 2a-c). Figure 2d shows that compared with that of the control group, the invasion and migration rates of the miR-15b-3p mimics-transfected SGC-7901 and BGC-823 cells were significantly higher, whereas the miR-15b-3p inhibitor alone transfected cells could only migrate or invade a short distance. TUNEL assay and flow cytometry analysis were used to explore whether the regulation of apoptosis is a potential factor for miR-15b-3p-induced cell growth progression. Thus, the apoptotic percentage of miR-15b-3p-silenced GC cells were found to be obviously elevated, and the cells overexpressing miR-15b-3p were found to show lower apoptosis levels (Fig. 2e and f). In addition, apoptosis-related protein expression levels were ascertained using western blotting analysis, as shown in Fig. 2g. Significant upregulation of the anti-apoptotic protein, BCL-2, expression was detected in the miR-15b-3p mimics group, which is contrary to that of BAX, Cleaved caspase-9 and Cleaved caspase-3 levels. The above mentioned changes were found to be opposite to the changes seen in the miR-15b-3p inhibition group. Accordingly, we speculate that miR-15b-3p functions as an oncogene in GC.

### MiR-15b-3p directly targets DYNLT1

**Fig. 3:**
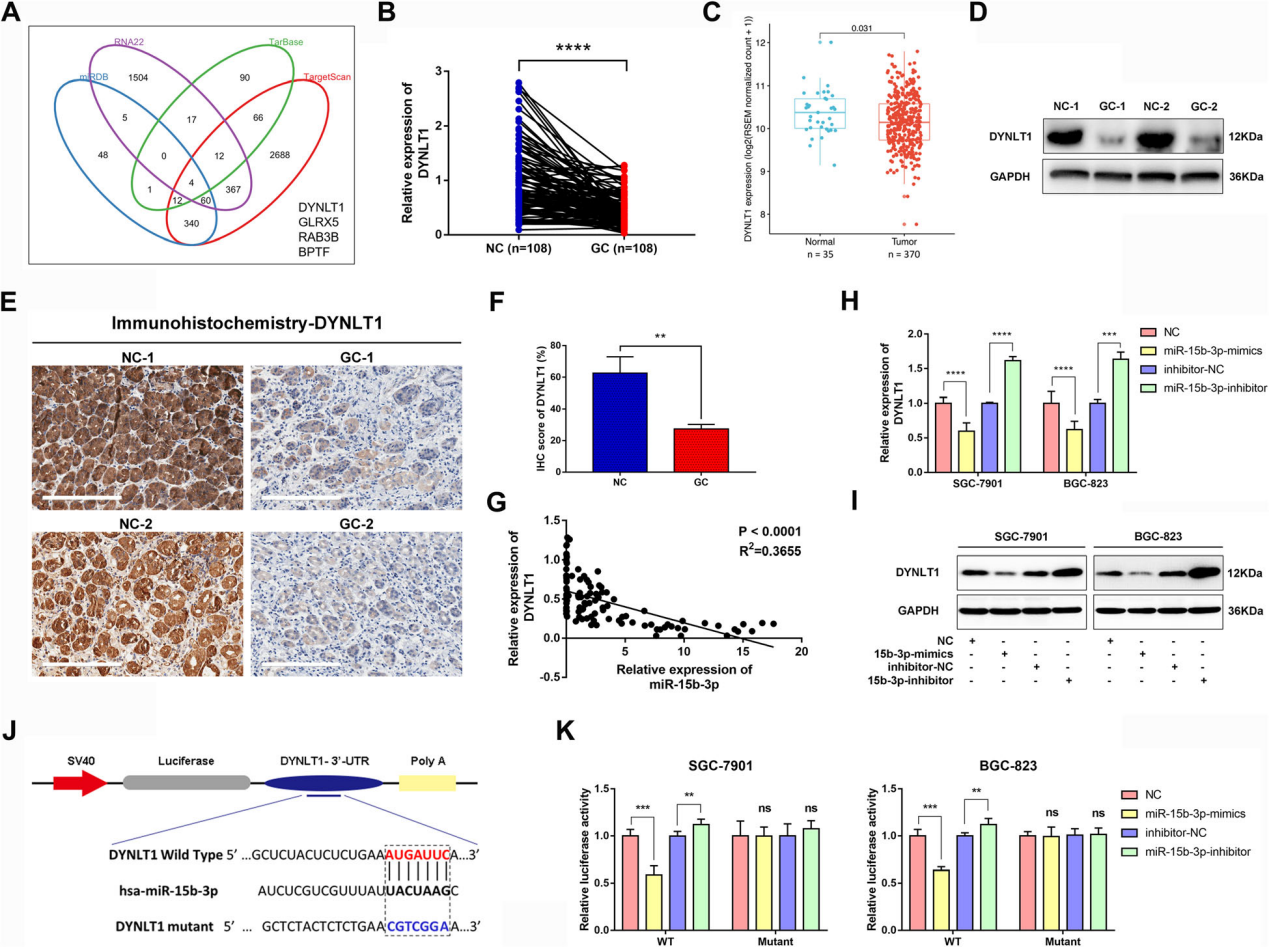
DYNLT1 is a direct downstream miR-15b-3p target in GC cells. **a** miR-15b-3p target gene prediction using four bioinformatics tools (miRDB, RNA22, TarBase and TargetScan). **b** DYNLT1 mRNA levels in GC tissues and paired adjacent non-GC tissues (*n* = 108) analyzed using qRT-PCR. **c** GC tissue DYNLT1 expression was found to be significantly decreased, based on the TCGA database. **d-f** Western blotting and IHC analysis of DYNLT1 protein levels in GC tissues and adjacent non-GC tissues. Scale bar, 200 μm. **g** miR-15b-3p and DYNLT1 expression level association analysis using the 108 GC tissues. **h-i** Immunoblotting assays and qRT-PCR on DYNLT1 expression of miR-15b-3p inhibitor/inhibitor-NC/mimics/NC transfected BGC-823 and SGC-7901 cells. **j** DYNLT1 binding site of the wild-type (WT) and mutated type with miR-15b-3p. **k** Direct recognition of DYNLT1 3′-UTR by miR-15b-3p. Co-transfection of BGC-823 and SGC-7901 cells with WT or Mutant DYNLT1 3′-UTR and miR-15b-3p mimics, inhibitor or their corresponding normal control (NC or inhibitor-NC). The relative luciferase activity of BGC-823 and SGC-7901 cells were determined. Mean ± SEM of the results are presented

miRNAs play a key role as negative gene expression regulators at post-transcription level by fusing target mRNA complementary 3’UTR sequences [13, 16]. In order to explore miR-15b-3p regulation at mRNA level, prediction of potential miR-15b-3p target genes was done by simultaneously using four bioinformatics tools (miRDB, RNA22, TarBase and TargetScan) (Fig. 3a). A significant decrease in DYNLT1 at mRNA level was found in GC tissues, as shown by qRT-PCR assays (Additional file 3: Figure S3a-c and Fig. 3b). A similar trend was seen with DYNLT1 expression in TCGA database GC tissues and normal tissues (Fig. 3c). Determination of DYNLT1 expression was done using western blotting and immunohistochemistry (IHC) analyses. As predicted, the results were similar, indicating that in GC tissues, DYNLT1 is downregulated, compared with matched normal tissues (*n* = 30, Fig. 3d-f). The results of the qRT-PCR analysis performed on 108 paired GC tumor tissues (*R*^2^ = 0.3655, *P*<0.0001), SGC-7901 cells (*R*^2^ = 0.7726, *P* = 0.0008) and BGC-823 cells (*R*^2^ = 0.8703, *P*<0.0001) found a negative correlation between the expressions of miR-15b-3p and DYNLT1 (Fig. 3g and Additional file 4: Figure S4). In addition, miR-15b-3p was confirmed to downregulate DYNLT1 expression in BGC-823 and SGC-7901 cells at both mRNA, as well as protein level, while DYNLT1 expression upregulation could be achieved by silencing miR-15b-3p (Fig. 3h and i). The direct interaction between miR-15b-3p and DYNLT1 was demonstrated using either a wild-type (WT) or mutant 3′-UTR DYNLT1 mRNA containing a luciferase reporter plasmid. As shown in Fig. 3j, miR-15b-3p harbors a complementary binding sequence of DYNLT1. Subsequently, miR-15b-3p overexpressing BGC-823 and SGC-7901 cells showed a significant decrease in luciferase activity, whereas the luciferase activity was obviously enhanced by miR-15b-3p inhibition (Fig. 3k). However, the loss of binding sites eliminated the miR-15b-3p inhibitory effect on luciferase activity, as shown in Fig. 3k. Thus, DYNLT1 was confirmed as a direct downstream miR-15b-3p target.

### Serum exo-miR-15b-3p as a potential GC diagnosis and prognosis biomarker

**Fig. 4:**
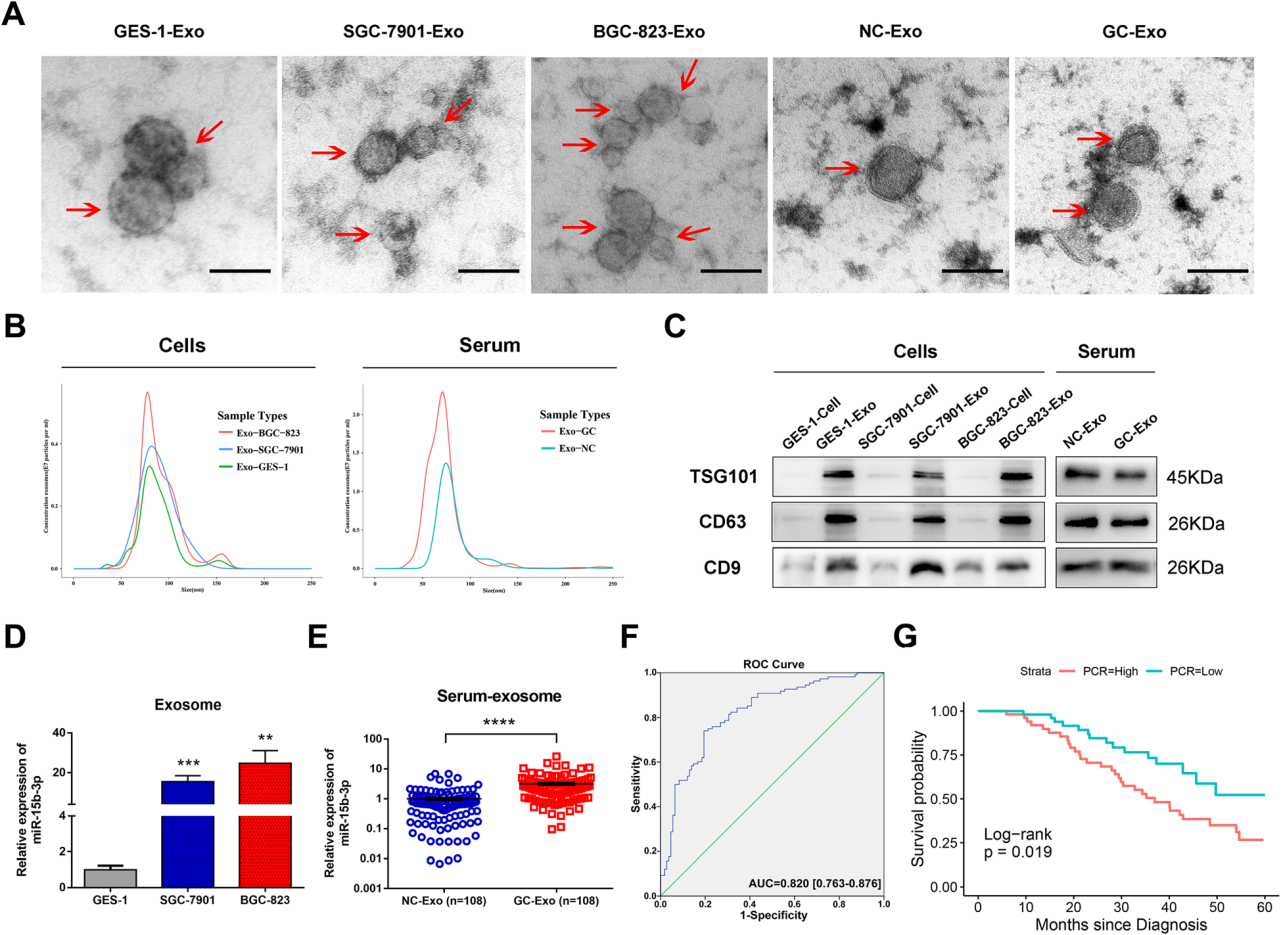
Exo-miR-15b-3p high expression in GC patient serum as a GC diagnosis and prognosis biomarker. **a** Representative electron microscopy micrographs of SGC-7901, BGC-823 and GES-1 cell conditioning medium secreted exosomes, as well as control and GC patient serum secreted exosomes. Scale bar, 100 nm. **b** Nano Sight particle-tracking analysis to determine exosome size distribution and number. **c** Levels of exosomal markers, TSG101, CD63 and CD9, of cells and serum derived exosomes determined using western blotting analysis. **d** Relative expressions of Exo-miR-15b-3p in conditioned medium of BGC-823, SGC-7901 and GES-1 cells. **e** Relative exosomal miR-15b-3p levels in GC patients and non-GC normal volunteer (*n* = 108) serum. **f** The sensitivity and specificity of serum Exo-miR-15b-3p for GC prediction was evaluated through Receiver-operating characteristic (ROC) curve analysis. **g** The correlation between serum Exo-miR-15b-3p expression and overall survival of the 108 GC patients determined using Kaplan–Meier analysis. The cutoff used was the median. Mean ± SEM of the results are presented

Considering that exosomes are highly stable disease biomarkers, exo-miRNAs may be potential GC diagnostic or prognostic biomarkers that are more accurate and stable than miRNAs [26–28]. In order to explore whether exo-miR-15b-3p performs the above functions, we first extracted and purified exosomes from the conditioned media of three cell lines (BGC-823, SGC-7901 and GES-1) and the serum of GC patients and non-GC volunteers (*n* = 108, patients and volunteers as listed in Table 1). Exosome quantity and number, as well as their cup-shaped morphology were determined through TEM analysis and Nano Sight particle tracking analysis (Fig. 4a and b). Moreover, the exosomal markers, TSG101, CD63 and CD9, were identified using western blotting analysis, further confirming that exosomes were the particles that were isolated (Fig. 4c). exo-miR-15b-3p was found to be enriched in the CM of SGC-7901 cells, and in particular, BGC-823 cells, rather than GES-1 cells, as shown by the qRT-PCR assay (Fig. 4d). Similarly, miR-15b-3p expression in the 108 GC patient serum exosomes was found to be significantly higher, compared with that of corresponding non-GC controls (Fig. 4e). exo-miR-15b-3p diagnostic efficacy for GC was determined using the ROC curve. The results show that the AUC of 0.820 (95%CI, 0.763–0.876), with a specificity of 80.6% and a sensitivity of 74.1% was obtained for exo-miR-15b-3p, as shown in Fig. 4f. The diagnostic effect of serum exo-miR-15b-3p was found to be better than that of miR-15b-3p in tissues (AUC = 0.674 [0.600–0.748]) and serum (AUC = 0.642 [0.499–0.786], Additional file 5: Figure S5a and b). The correlation between clinicopathological features and serum exo-miR-15b-3p levels of expression were determined by dividing patients into a high-expression group and a low-expression group, with 54 patients assigned to each, based on the median miR-15b-3p expression level. A statistically significant correlation was observed between high serum exo-miR-15b-3p expression and alcohol abuse, tumor size (≥3.5 cm in diameter), poorly differentiated histological type, TNM stage (III and IV) and lymph vascular invasion (Table 2). Furthermore, Kaplan-Meier analysis was performed to judge whether serum exo-miR-15b-3p expression is correlated with GC patient cancer-specific survival. As shown in Fig. 4g, poor overall survival (*P* = 0.019) can be accurately predicted by high exo-miR-15b-3p expression levels. These results indicate that serum-secreted exo-miR-15b-3p can function as a sensitive and specific predictive and prognostic liquid biomarker for GC and may be related with the malignant transformation of GC.

**Table 2:** Association of miR-15b-3p Expression in GC with Different Clinicopathological Features

Variables	Exo-miR-15b-3p expression		*P-*value
	Low(*n* = 54)	High(*n* = 54)	
Age, years			0.425
<60	22(40.7%)	18(33.3%)	
≥ 60	32(59.3%)	36(66.7%)	
Gender			0.661
Male	41(75.9%)	39(72.2%)	
Female	13(24.1%)	15(27.8%)	
Smoking			0.311
Yes	16(29.6%)	21(38.9%)	
No	38(70.4%)	33(61.1%)	
Alcohol abuse			0.024*
Yes	8(14.8%)	18(33.3%)	
No	46(85.2%)	36(66.7%)	
Family history of cancer			0.206
Yes	7(13.0%)	12(22.2%)	
No	47(87.0%)	42(77.8%)	
Size, cm			0.020*
<3.5	30(55.6%)	18(33.3%)	
≥ 3.5	24(44.4%)	36(66.7%)	
Location			0.267
Cardia	16(29.6%)	11(20.4%)	
Non-cardia	38(70.4%)	43(79.6%)	
Lauren’s classification			0.101
Intestinal type	22(40.7%)	14(25.9%)	
Diffuse type	20(37.0%)	31(57.4%)	
Mixed type	12(22.2%)	9(16.7%)	
Histological grade			0.011*
Well differentiated	22(40.7%)	10(18.5%)	
Poor differentiated	32(59.3%)	44(81.5%)	
Invasion depth			0.176
T1/2	28(51.9%)	21(38.9%)	
T3/4	26(48.1%)	33(61.1%)	
Tumor stage			0.002*
I,II	38(70.4%)	22(40.7%)	
III,IV	16(29.6%)	32(59.3%)	
Lymphovascular invasion			0.001*
Negative	37(68.5%)	20(37.0%)	
Positive	17(31.5%)	34(63.0%)	

*P < 0.05

### Transfer of miR-15b-3p from BGC-823 cell-derived exosomes to recipient cells

**Fig. 5:**
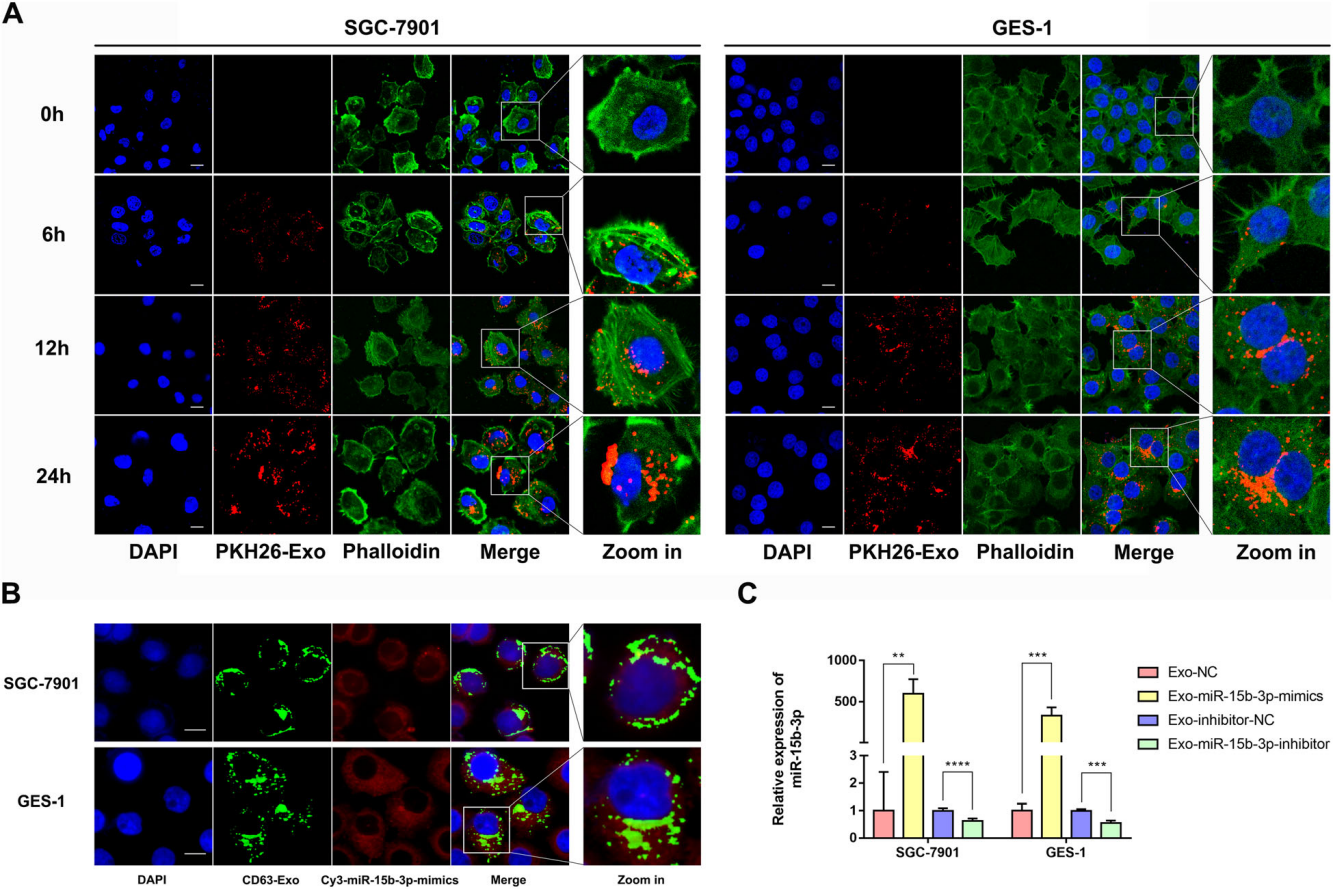
Exosome-mediated miRNA transport between cells. **a** Internalization of PKH26-labelled exosomes (red) in GES-1 and SGC-7901 cells were observed through confocal microscopy. Fluorescein phalloidin-FITC (green) was used to stain F-actin, while DAPI (blue) was used to stain nuclei. Scale bar, 20 μm. **b** Exosomes (green) isolated from BGC-823 cell conditioning medium labeled with GFP-Lv-CD63 (green) and transfected with Cy3-miR-15b-3p (red) were co-cultured with SGC-7901 and GES-1 cells for 24 h, and the fluorescent signals were detected using confocal microscopy. Nuclei are stained blue (DAPI). Scale bar, 20 μm. **c** The efficiency of exosomes in delivering miR-15b-3p to GES-1 and SGC-7901 cells was analyzed using RT-PCR. Mean ± SEM of the results are presented

Since miR-15b-3p expression of BGC-823 cells (poorly differentiated adenocarcinoma) is higher than that of SGC-7901 and GES-1 cells, we hypothesized that exosomes can mediate a novel mechanism of cell-to-cell communication in GC, by transmitting miR-15b-3p between cells of varying degrees of differentiation and malignancy, and then take part in the malignant transformation of GC. In order to confirm our assumption and the manner of miR-15b-3p intercellular delivery, we performed co-culture experiments to determine if exosomes and their contents could be internalized by target cells. First, 50 mg of BGC-823 cell derived PKH26-labeled exosomes were incubated with 5 × 10^5^ GES-1 or SGC-7901 cells, and the uptake of exosomes was observed after co-culture for 0, 6, 12 and 24 h. It was found that in a time-dependent manner, GES-1 and SGC-7901 cells gradually engulfed the exosomes (Fig. 5a). After 24 h of co-cultivation, many exosomes were found to have entered recipient cells and accumulated around the nucleus (Fig. 5a). Moreover, in order to visualize the exosome-mediated intercellular miRNA transfer, after confirming that GFP-Lv-CD63 had been successfully transfected into BGC-823 cells (Additional file 6: Figure S6a), the fluorescence labeled miR-15b-3p mimics (Cy3-miR-15b-3p mimics) was transiently transfected into BGC-823 cells (Additional file 6: Figure S6b) and then, the medium was refreshed (Additional file 6: Figure S6c). Next, exosomes in the CM of transfected BGC-823 cells were further isolated and added to the untreated GES-1 and SGC-7901 cells for 24 h. The apparent green and red fluorescence seen in Fig. 5b confirms the successful shuttling of Cy3-miR-15b-3p mimics through exosomes into recipient cells. Moreover, Cy3-miR-15b-3p mimics and CD63-labeled exosomes were found to be co-localized in the cytoplasm (Fig. 5b). In addition, the qRT-PCR assay results show that oligonucleotide sequences (miR-15b-3p mimics/NC/inhibitor/inhibitor-NC) can be taken-up by exosomes and transported into the extracellular medium, where recipient cell uptake regulates miR-15b-3p expression (Fig. 5c).

### Intercellular transfer of miR-15b-3p by exosomes promote malignant transformation in vitro

**Fig. 6:**
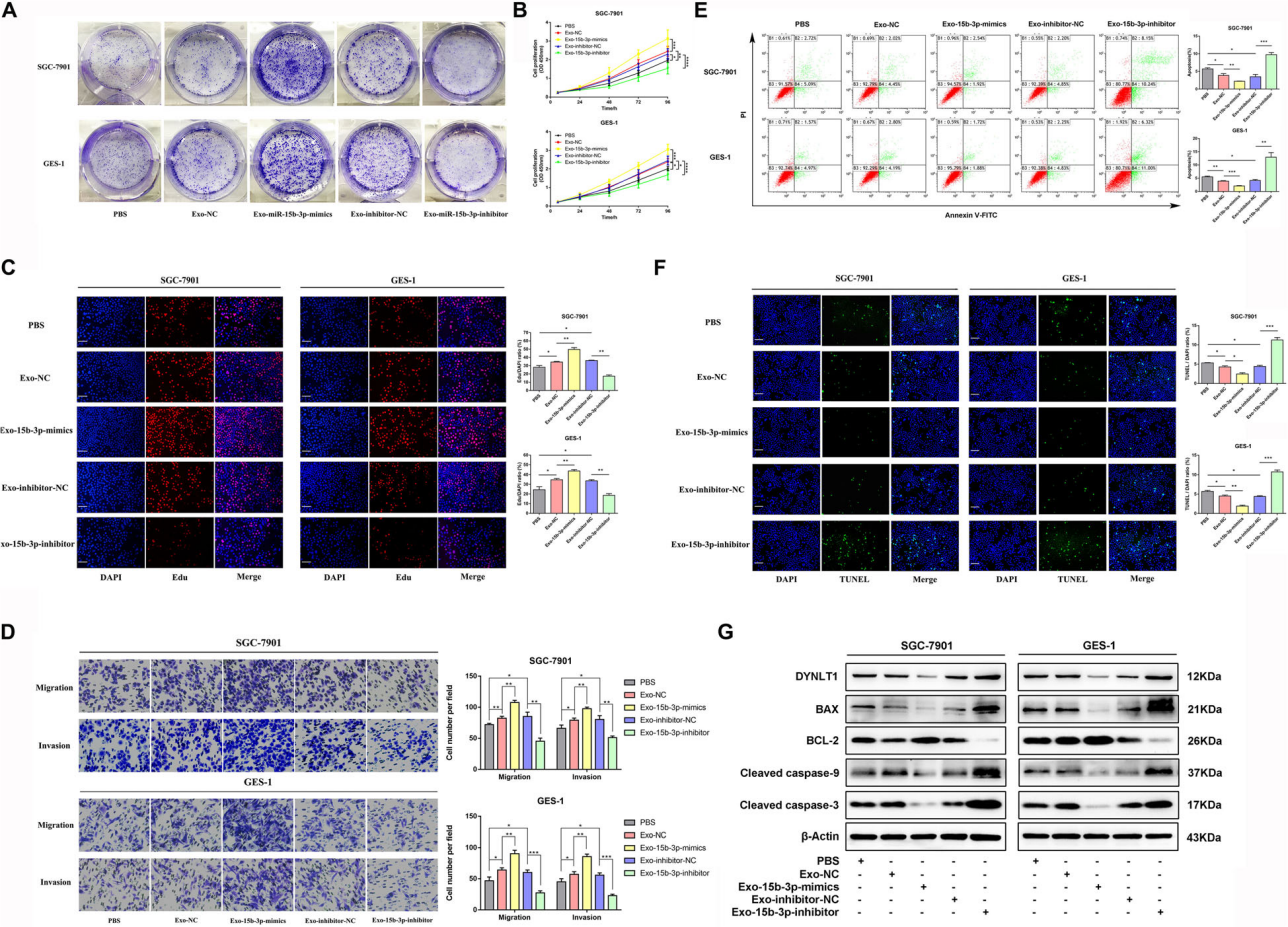
Exosomal transfer of miR-15b-3p enhances malignant transformation in vitro. Proliferation of SGC-7901 and GES-1 cells co-cultured with PBS alone or exosomes containing miR-15b-3p mimics/NC/inhibitor/inhibitor-NC were assessed using colony formation (**a**), CCK-8 (**b**) and EdU (**c**) assays. Scale bar, 100 μm. **d** Migration and invasion assays of SGC-7901 and GES-1 cell treated with PBS, Exo-NC, Exo-15b-3p-mimics, Exo-inhibitor-NC or Exo-15b-3p-inhibitor. Cells that had migrated and invaded were counted. Representative images are shown. **e** The SGC-7901 and GES-1 cell apoptosis, in the presence of PBS or exosomes (wrapped with 15b-3p-mimics, inhibitor or their corresponding normal control) were detected using flow cytometry. **f** miR-15b-3p mimics/NC/inhibitor/inhibitor-NC-enriched exosomes or only PBS was incubated with GES-1 and SGC-7901 cells for 24 h, followed by TUNEL assay. **g** Western blotting assay of DYNLT1, BAX, BCL-2, Cleaved Caspase-9 and Cleaved Caspase-3 in SGC-7901 and GES-1 with the treatments indicated. The internal control used was β-Actin. Mean ± SEM of three independent experiments are presented

For further investigating BGC-823 cell-derived exo-miR-15b-3p function in recipient cells, we isolated exosomes from the CM of miR-15b-3p inhibitor/inhibitor-NC /mimics/NC transfected BGC-823 cells. Next, 50 mg of the purified exosomes or PBS was co-cultured for 24 h with 5 × 10^5^ GES-1 or SGC-7901 cells. As expected, when exo-miR-15b-3p mimics were incubated with either SGC-7901 or GES-1 cell lines, increased cell proliferation (tested using colony formation, CCK-8 and EdU assays) (Fig. 6a-c), cell invasion and migration (tested using Transwell chamber migration assay) (Fig. 6d) were observed. In contrast, significant repression of these biological functions were found in cells co-cultured with exosomes, in which miR-15b-3p was knocked down (Fig. 6a-d). However, recipient cells treated with control exosomes (Exo-NC and Exo-inhibitor-NC) showed greater proliferation, migration and invasion capacities than those treated with PBS (Fig. 6a-d). Furthermore, we found that the apoptosis of GES-1 and SGC-7901 cells treated with exosomes carrying miR-15b-3p mimics was significantly reduced, while knockdown of exo-miR-15b-3p reversed the apoptosis of these cells (Fig. 6e-g). At the protein level, the expression of DYNLT1 and BAX were found to be inhibited in GES-1 and SGC-7901 cells treated with exo-miR-15b-3p mimics, compared with Exo-NC and PBS groups. Conversely, transfection with exosomes containing a miR-15b-3p inhibitor had an opposite effect on their expression (Fig. 6g). In addition, anti-apoptotic protein BCL-2 levels increased in GES-1 and SGC-7901 cells co-incubated with exosomes packed with miR-15b-3p mimics and decreased in cells treated with the exo-miR-15b-3p inhibitor (Fig. 6g). It is known that DYNLT1 is involved in the regulation of apoptosis [29, 30]. In order to further explore its mechanism of action, we analyzed levels of major proteins in the Caspase-3 classical apoptosis signaling pathway and found increased cleavage of Caspase-9 and Caspase-3 in the high DYNLT1 expression group, which was inhibited in the low DYNLT1 expression group (Fig. 6g). Collectively, these results indicate that BGC-823 cell-derived exo-miR-15b-3p is effectively involved in the malignant transformation of recipient cells.

### Exo-miR-15b-3p/DYNLT1/Caspase-3/Caspase-9 enhances tumorigenicity in vivo

**Fig. 7:**
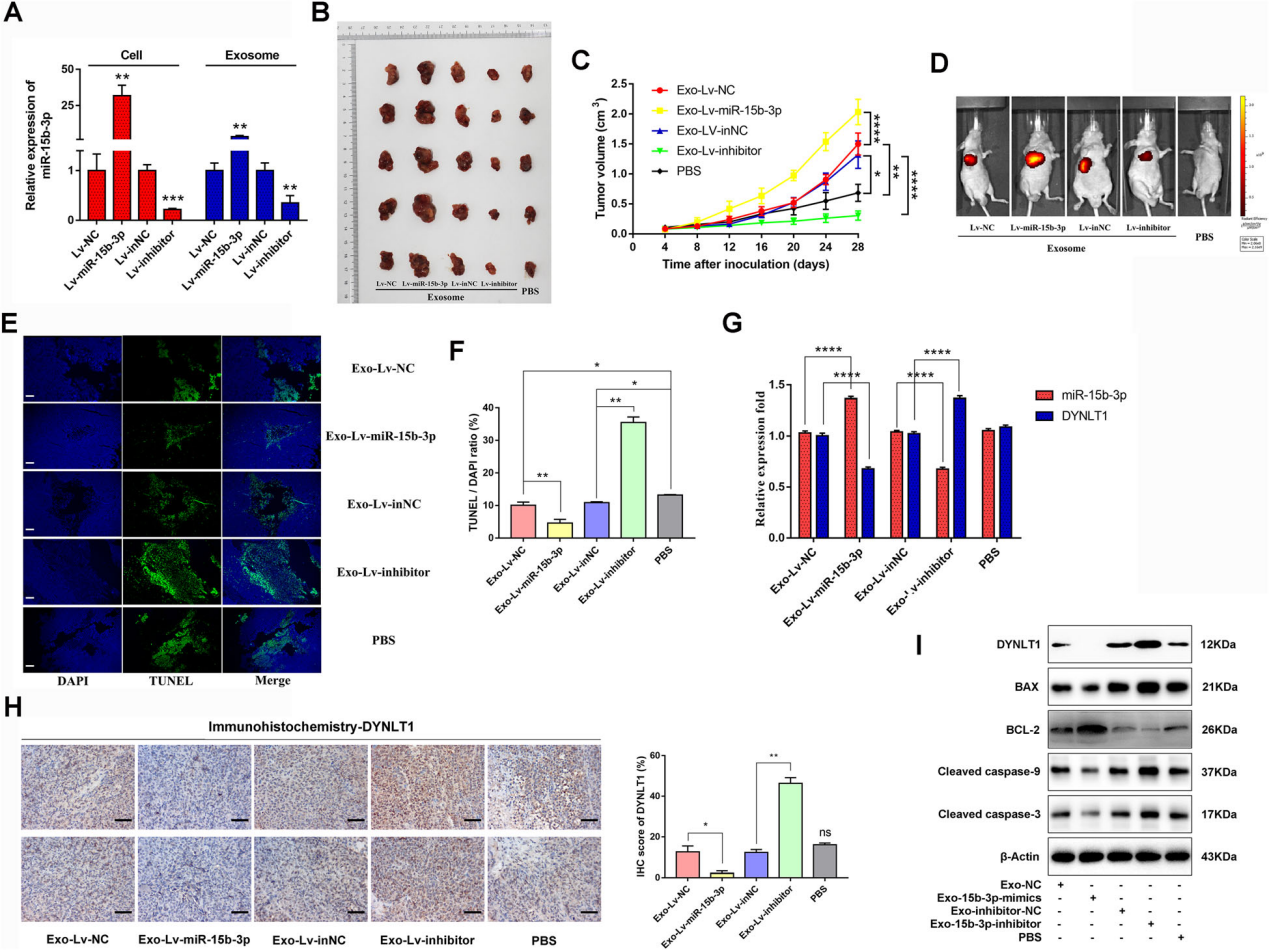
Exo-miR-15b-3p regulates tumor growth in vivo. **a** miR-15b-3p expression levels in BGC-823 cells (stably transfected with Lv-miR-15b-3p/Lv-NC or Lv-inhibitor/Lv-inNC) or exosomes isolated from BGC-823 cells were detected using qRT-PCR. SGC-7901 cells were treated with PBS or exosomes loaded with Lv-miR-15b-3p/Lv-NC or Lv-inhibitor/Lv-inNC and were subsequently injected into the nude mice (*n* = 5). The xenografts (**b)** and tumor growth curve (**c)** show that Exo-Lv-miR-15b-3p promotes, while Exo-Lv-inhibitor suppresses xenograft tumor growth in nude mice. **d** Representative images of tumor growth of the mice treated with exosomes derived from stably transfected-BGC-823 cells or PBS, were determined using luciferase-based bioluminescence imaging. **e** Representative images of TUNEL staining of the xenograft tumors for the ectopic expression or silencing of Exo-miR-15b-3p and their corresponding control or PBS groups. Scale bar, 100 μm. **f** Quantification of TUNEL-positive cells. **g** qRT-PCR analysis of miR-15b-3p and DYNLT1 expression in xenograft tumors with the treatment indicated. **h** Immunohistochemical analysis of DYNLT1 expression in the xenografts. Scale bar, 50 μm. **i** Western blotting analysis of DYNLT1, BAX, BCL-2, Cleaved Caspase-9 and Cleaved Caspase-3 in xenograft tumor tissues among the different groups. The internal control used was β-Actin. Mean ± SEM of the results are presented

Subsequently, the in vitro changes seen in GES-1 and GC cells in the presence of exo-miR-15b-3p were confirmed in vivo. The nude mice were subcutaneously injected with SGC-7901 cells, following BGC-823 cell-derived Exo-Lv-NC, Exo-Lv-miR-15b-3p, Exo-Lv-inNC and Exo-Lv-inhibitor or PBS pre-incubation. The stable transfection efficiency of the luciferase-labelled lentivirus into BGC-823 cells and the miR-15b-3p expression of exosomes derived from cells of different treatment groups are shown in Fig. 7a. We observed that tumor growth increased significantly in mice receiving exosomes enriched with miR-15b-3p, compared with those injected with PBS or exosomes containing Lv-NC (Fig. 7b and c), at the same time luciferase intensities were also detected (Fig. 7d). However, visibly smaller tumors were formed in the Exo-Lv-inhibitor group (Fig. 7b-d). Next, tumor tissues harvested for TUNEL staining were found to show a decrease in GC cell apoptotic rate, after treatment with Exo-Lv-miR-15b-3p, compared with those treated with Exo-Lv-NC, whereas the opposite result was detected in the Exo-Lv-inhibitor group (Fig. 7e and f). Furthermore, we found the results to be consistent with the in vitro results, indicating that both exosome-delivered NC and inhibitor-NC significantly repressed cell apoptosis, compared with treatment with PBS alone, suggesting that BGC-823 cell-derived exosomes can inhibit target cell apoptosis (Fig. 7e and f). Compared with the control group, qRT-PCR quantified tumor tissue miR-15b-3p levels were significantly higher in the Exo-Lv-miR-15b-3p group and decreased in the Exo-Lv-inhibitor group, while the results of both qRT-PCR and IHC assays indicate that DYNLT1 expression in tissues is inhibited in the first group and enhanced in the second group (Fig. 7g and h). However, as shown in Fig. 7g and h, no obvious difference was found in the PBS group. Exo-Lv-miR-15b-3p treated tumors with high miR-15b-3p levels tended to express lower protein levels of DYNLT1, BAX, Cleaved caspase-9 and Cleaved caspase-3, but higher protein levels of BCL-2 (Fig. 7i). Conversely, higher protein levels of DYNLT1, BAX, Cleaved caspase-9 and Cleaved caspase-3, but lower BCL-2 levels were observed in the low miR-15b-3p groups (Exo-Lv-inhibitor treated) (Fig. 7i). Our results suggest that the exo-miR-15b-3p/DYNLT1 axis inhibits apoptosis via modulating the Caspase-3/Caspase-9 signaling pathway, maintaining high levels of SGC-7901 cell proliferation in vivo.

## Discussion

Evidence from previous research has provided the following sequential stages for a human gastric carcinogenesis model: chronic active gastritis, gastric atrophy, intestinal metaplasia, and dysplasia [31]. The development of tumors, including GC, requires continued oncogenic reprogramming to determine the malignant characteristics of cells. Exosomes are potential communicative vectors that act as intercellular mediators, providing dual-role oncosignals in gastric tumorigenesis [32]. Among them, carcinogenic components of GC-derived exosomes can cause the malignant transformation of recipient cells, promoting cell proliferation and migration [33–35]. It has been found that tumor progression and growth can be successfully analyzed by studying exosomes. Currently, exo-miRNAs can regulate different pathological and physiological processes through the inhibition or activation of certain regulatory pathways by shuttling into recipient cells and modifying gene or protein expression, especially for the regulation of GC processes. Thus, serving as circulating biomarkers of GC and a tool for targeted therapies [8, 13, 32, 36, 37].

As important gene regulators, the miR-15b family is involved in the cell cycle, cellular proliferation and apoptosis, and has been found to be dysfunctional in various diseases [38]. Expression levels of miR-15b-3p have been reported to be significantly upregulated in rs363050 SNAP-25 GG homozygous Alzheimer’s disease [39], Microcystin-LR-induced hepatotoxicity [40], myocardial ischemic reperfusion injury [41], coronary artery disease [42], and poor prognosis of hepatocellular carcinoma patients after curative hepatectomy [43]. Therefore, miR-15b-3p expression may be positively correlated with the progression of the disease. Moreover, serum miR-15b-3p levels have been reported to constitute a novel biomarker of epicardial fat burden [44], while serum miR-15b has potential as a predictive biomarker of obesity [45]. However, the potential association between the miR-15b family and GC is controversial. miR-15b has been shown to be downregulated in SGC7901/DDP cells [46] and gastric adenoma [47], while Yuan et al. [48] has shown the significant overexpression of miR-15b in GC, which was found by analyzing 1000 GC samples included in four public datasets. In addition, miR-15b-5p impact on invasion, migration and proliferation of GC cells with high miR-15b-5p levels in GC cell lines, tissues and serum samples were confirmed by Zhao et al. [49]. In addition, miR-15b has been repeatedly demonstrated to target important BCL-2 family proteins, including both anti-apoptotic (e.g., Bcl-2) and pro-apoptotic (e.g., Bax) members and regulate the expression of caspases 3, 7, 8, or 9, as well as participate in tumorigenesis and tumor development by enhancing or inhibiting cell activity, proliferation and apoptosis [50–55]. However, in GC, miR-15b-3p expression and function are not clear as yet.

The present study screened 13 miRNAs that may be involved in GC progression from among 29 miRNAs that were upregulated, in both the GSE86226 dataset and TCGA database using qRT-PCR analysis, in which miR-15b-3p was most overexpressed in GC tissues. miR-15b-3p overexpression was subsequently found in GC serum and cell lines for the first time. The key regulatory effects of miR-15b-3p on GC cell apoptosis have been confirmed by three different experimental methods. Consistently, we observed that GC cell miR-15b-3p overexpression increases BCL-2 expression, as well as decreases BAX, Cleaved caspase-9 and Cleaved caspase-3 expression, whereas miR-15b-3p knockdown reverses this effect. Thus, our results prove for the first time that miR-15b-3p is significantly upregulated in GC and acts as an oncogene for GC.

Moreover, miR-15b-3p was found to function directly by targeting DYNLT1, herein, its official complete name, dynein light chain Tctex-type 1, which is also known as CW-1, TCTEL1 or tctex-1. DYNLT1 encodes a component of the motor complex that transports cellular cargo along microtubules of the cell. Therefore, this gene may be an indispensable host cell protein for transporting material into the nucleus [56]. Meanwhile, the DYNLT1 gene located at 6q25.3 [57], the long arm of chromosome 6 (6q), has been found to be frequently lost in GC, especially in gastric adenocarcinoma [58–61], and may therefore harbor a tumor suppressor gene [61], which is consistent with the downregulation of DYNLT1 expression in GC found here. However, the effect of DYNLT1 on the progression of GC remains unclear.

In order to explore whether miRNAs are enriched and stable in the circulatory exosomal system, as previously reported [28], in the CM of GC cells and serum of 108 GC patients, exo-miR-15b-3p was found to be evidently overexpressed and can function as a potential GC diagnosis and poor prognosis biomarker. Moreover, we confirmed for the first time that exo-miR-15-3p is secreted by poorly differentiated adenocarcinoma (BGC-823) cells that can be internalized and absorbed by normal GES-1 gastric mucosa epithelium cells and moderately differentiated adenocarcinoma (SGC-7901) cells, suggesting that miR-15b-3p is probably suitable to be packed into exosomes to maintain its stability and intercellular transfer. A series of functional experiments that were subsequently conducted, demonstrated that exo-miR-15b-3p maintains miR-15b-3p carcinogenesis and is involved in tumorigenesis and GC progression, both in vivo and in vitro. This effect may be achieved by the exo-miR-15b-3p-induced downregulation of DYNLT1. Voltage-dependent anion channel 1 (VDAC1) is a key component of mitochondria-mediated apoptosis, and exerts a protective effect on anti-apoptotic proteins, including BCL-2 [62, 63]. Combined with the report by Ochiai et al. that DYNLT1 is the target protein of VDAC1 [30], we speculated that DYNLT1 is implicated in apoptosis regulation. In addition, DYNLT1 has previously been considered as an interacting partner of REIC/Dkk-3, inducing apoptosis through its action as a multiple cancer cell line tumor suppressor [29]. In our study, pro-apoptotic protein, BAX expression was found to be positively correlated with DYNLT1 expression, while the anti-apoptotic protein, BCL-2 expression showed an opposite trend to that of DYNLT1, and the Caspase-3/Caspase-9 pathway was subsequently activated to varying degrees along with changes in exo-miR-15b-3p-induced DYNLT1 expression. However, the precise mechanism by which DYNLT1 modulates the expression of the BCL-2 family of proteins and Cleaved Caspase-3/Caspase-9 signaling pathway activation are not clear, and we intend to explore these topics in future studies.

## Conclusion

**Fig. 8:**
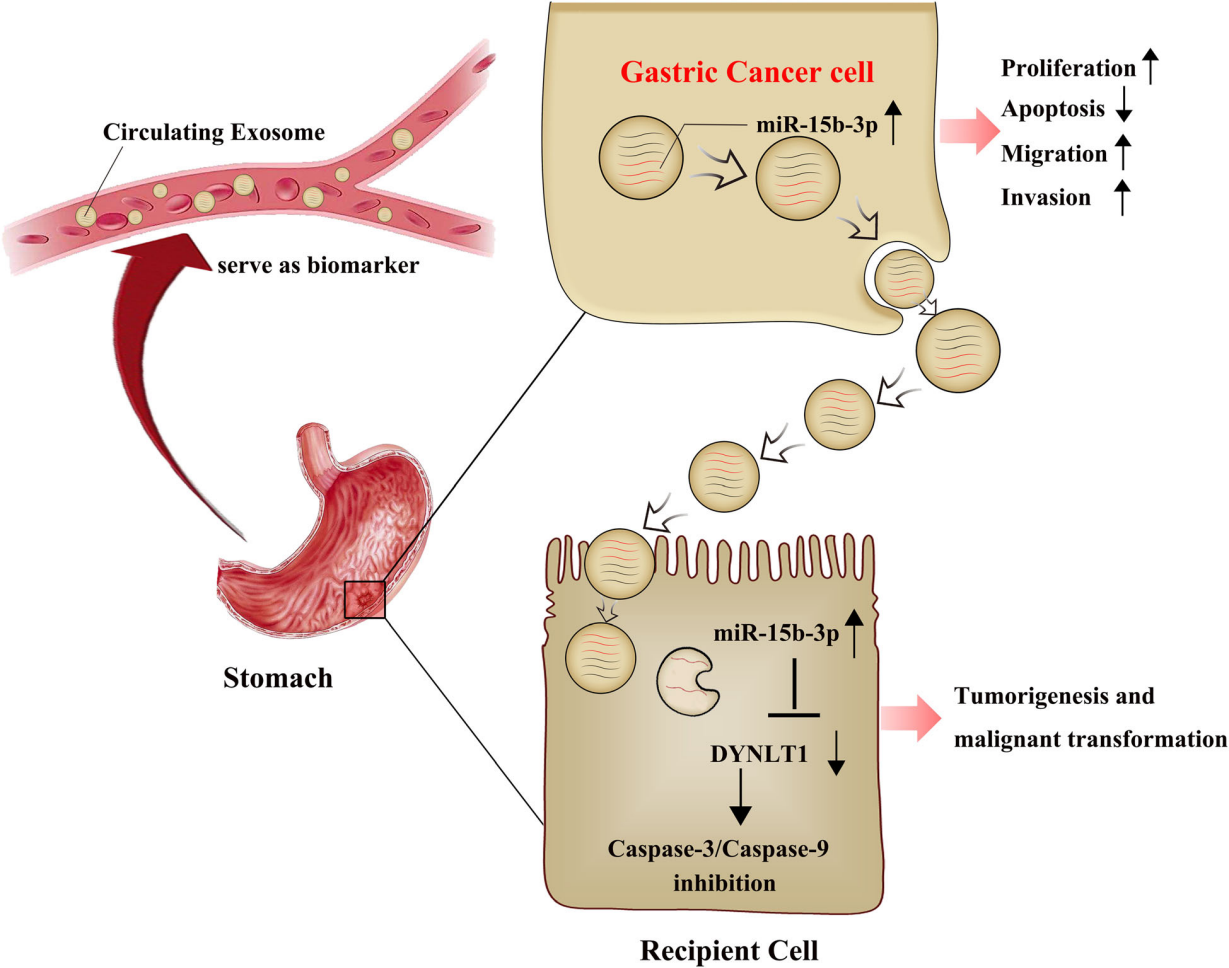
Schematic illustrating the function and mechanism of GC cells-derived exo-miR-15b-3p in recipient cells. The exo-miR-15b-3p released by GC cells promotes the proliferation, migration, invasion and inhibits apoptosis of recipient cells via the DYNLT1/Caspase-3/Caspase-9 signaling pathway, thereby promoting tumorigenesis and malignant transformation. Serum exo-miR-15b-3p may function as a potential GC diagnostic biomarker

In brief, our findings demonstrate for the first time that exosomes secreted by BGC-823 cells can transfer miR-15b-3p into recipient cells, promoting tumorigenesis and malignant transformation, as well as inhibiting apoptosis in vivo and in vitro via the DYNLT1/Caspase-3/Caspase-9 signaling pathway. Serum exo-miR-15b-3p in humans may function as a potential GC diagnostic and prognostic biomarker, acting as a significant novel GC therapeutic target (Fig. 8).

## Supplementary information

**Additional file 1: Figure S1.** Expression of miRNAs in 6 pairs of GC tissues and normal tissues. In addition to miR-15b-3p, 12 miRNAs that may play a role in GC cell proliferation and migration were screened out from among 29 potentially differentially expressed miRNAs. In order to evaluate the relative expression levels of miR-192-5p (a), miR-21-5p (b), miR-141-3p (c), miR-15b-5p (d), miR-185-5p (e), miR-532-5p (f), miR-331-3p (g), miR-106b-3p (h), miR-17-5p (i), miR-20a-3p (j), miR-501-3p (k) and miR-200c-3p (l), qRT-PCR assays were performed. The internal control used was U6. Mean ± SEM of the results are presented.

**Additional file 2: Figure S2. **a. qRT-PCR analysis of miR-15b-3p expression levels in SGC-7901 and BGC-823 cells after oligonucleotide transfection. The internal control was U6. Mean ± SEM of three independent experiments are presented.

**Additional file 3: Figure S3.** mRNA expression levels in 10 pairs of GC tissues and normal tissues. qRT-PCR analysis of GLRX5 (a), RAB3B (b) and BPTF (c) relative expression levels between GC tissue and paired adjacent non-GC tissue. The internal control was GAPDH. Mean ± SEM of the results are presented.

**Additional file 4: Figure S4.** The correlation between miR-15b-3p and DYNLT1 in vitro. Association analysis of the relationship between miR-15b-3p and DYNLT1 expression levels in SGC-7901 cells (a) and BGC-823 cells (b).

**Additional file 5: Figure S5**. ROC curves of tissue and serum miR-15b-3p in GC vs non-GC control groups. a. ROC curve of tissue miR-15b-3p panel to discriminate GC patients from NCs. **b.** ROC curves were used to determine the diagnostic efficacy of serum miR-15b-3p for GC. Mean ± SEM of the results are presented.

**Additional file 6: Figure S6.** Fluorescence images of BGC-823 cells after transfected. a Confocal microscopy images show that BGC-823 cells were stably transfected with GFP-Lv-CD63 (green). Scale bar, 25 μm. b. Fluorescence visuals of BGC-823 cells transfected with Cy3-miR-15b-3p mimics (red). Scale bar, 25 μm. **c** Red fluorescence was observed under fluorescence microscopy after refreshing the conditioned medium of the BGC-823 cells transfected with Cy3-miR-15b-3p mimics. Scale bar, 25 μm.

**Additional file 7: Table S1.** Real-time polymerase chain reaction primers. **Table S2.** Sequences of miR-15b-3p oligo.

## Data Availability

All data generated or analyzed during this study are included either in this article or in the additional files.

## Abbreviations

BCA: Bicinchoninic acid
DAPI: 4′6-diamidino-2-phenylindole
FITC: Fluorescein isothiocyanate
GAPDH: Glyceraldehyde 3-phosphate dehydrogenase
miR-15b-3p: microRNA-15b-3p
qRT-PCR: Reverse transcription quantitative polymerase chain reaction
RPMI: Roswell Park Memorial Institute
SEM: Standard error of the mean
SPSS: Statistical product and service solutions
