# Supplementary figures and images for: Exosomal transfer of miR-15b-3p enhances tumorigenesis and malignant transformation through the DYNLT1/Caspase-3/Caspase-9 signaling pathway in gastric cancer
Source: J Exp Clin Cancer Res. 2020 Feb 10;39:32. doi: 10.1186/s13046-019-1511-6 (PMC7011526; doi:10.1186/s13046-019-1511-6)

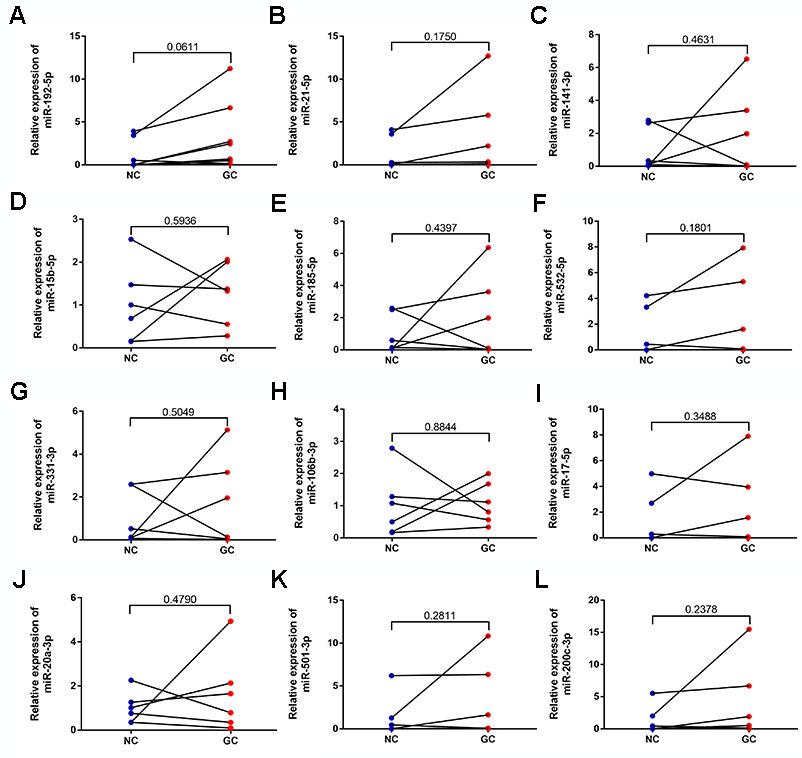

Supplement: Supplementary file 1 — Additional file 1: Figure S1. Expression of miRNAs in 6 pairs of GC tissues and normal tissues. In addition to miR-15b-3p, 12 miRNAs that may play a role in GC cell proliferation and migration were screened out from among 29 potentially differentially expressed miRNAs. In order to evaluate the relative expression levels of miR-192-5p (a), miR-21-5p (b), miR-141-3p (c), miR-15b-5p (d), miR-185-5p (e), miR-532-5p (f), miR-331-3p (g), miR-106b-3p (h), miR-17-5p (i), miR-20a-3p (j), miR-501-3p (k) and miR-200c-3p (l), qRT-PCR assays were performed. The internal control used was U6. Mean ± SEM of the results are presented. [file 13046_2019_1511_MOESM1_ESM.tif]

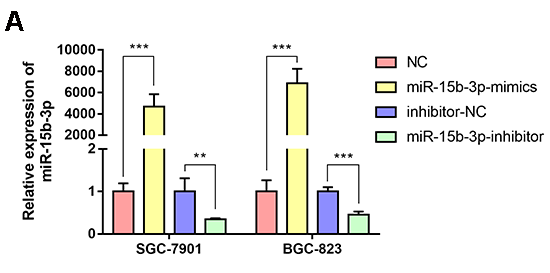

Supplement: Supplementary file 2 — Additional file 2: Figure S2. a. qRT-PCR analysis of miR-15b-3p expression levels in SGC-7901 and BGC-823 cells after oligonucleotide transfection. The internal control was U6. Mean ± SEM of three independent experiments are presented. [file 13046_2019_1511_MOESM2_ESM.tif]

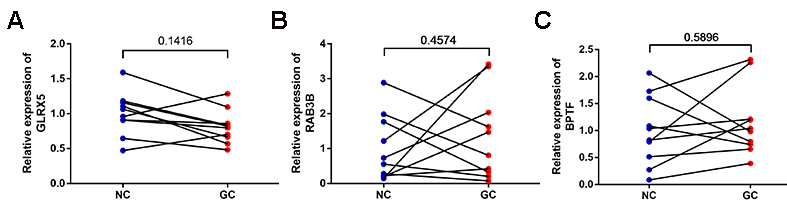

Supplement: Supplementary file 3 — Additional file 3: Figure S3. mRNA expression levels in 10 pairs of GC tissues and normal tissues. qRT-PCR analysis of GLRX5 (a), RAB3B (b) and BPTF (c) relative expression levels between GC tissue and paired adjacent non-GC tissue. The internal control was GAPDH. Mean ± SEM of the results are presented. [file 13046_2019_1511_MOESM3_ESM.tif]

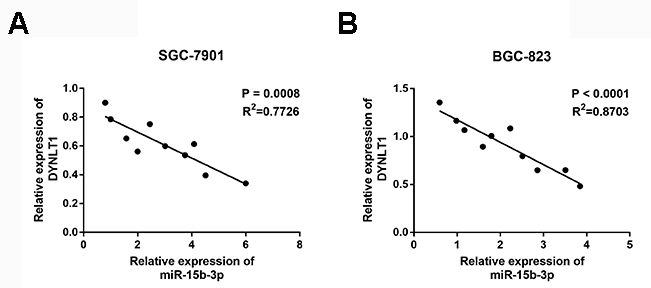

Supplement: Supplementary file 4 — Additional file 4: Figure S4. The correlation between miR-15b-3p and DYNLT1 in vitro. Association analysis of the relationship between miR-15b-3p and DYNLT1 expression levels in SGC-7901 cells (a) and BGC-823 cells (b). [file 13046_2019_1511_MOESM4_ESM.tif]

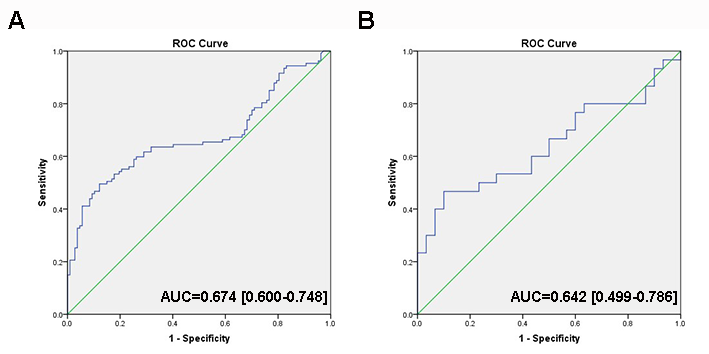

Supplement: Supplementary file 5 — Additional file 5: Figure S5. ROC curves of tissue and serum miR-15b-3p in GC vs non-GC control groups. a. ROC curve of tissue miR-15b-3p panel to discriminate GC patients from NCs. b. ROC curves were used to determine the diagnostic efficacy of serum miR-15b-3p for GC. Mean ± SEM of the results are presented. [file 13046_2019_1511_MOESM5_ESM.tif]

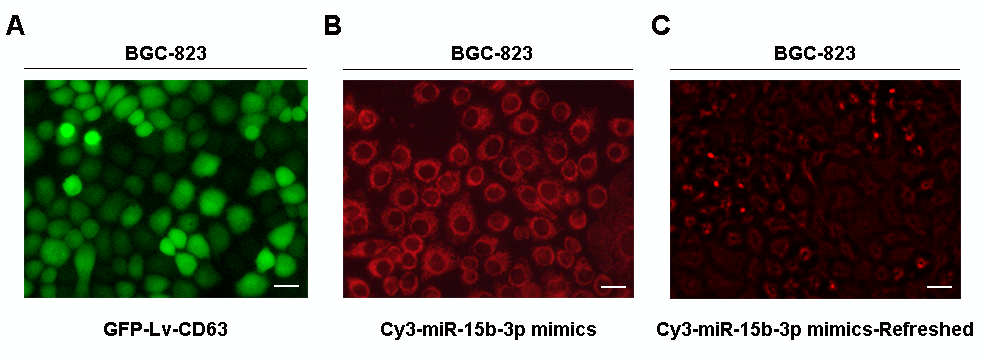

Supplement: Supplementary file 6 — Additional file 6: Figure S6. Fluorescence images of BGC-823 cells after transfected. a Confocal microscopy images show that BGC-823 cells were stably transfected with GFP-Lv-CD63 (green). Scale bar, 25 μm. b. Fluorescence visuals of BGC-823 cells transfected with Cy3-miR-15b-3p mimics (red). Scale bar, 25 μm. c Red fluorescence was observed under fluorescence microscopy after refreshing the conditioned medium of the BGC-823 cells transfected with Cy3-miR-15b-3p mimics. Scale bar, 25 μm. [file 13046_2019_1511_MOESM6_ESM.tif]
