# Supplementary material for: Exosomal transfer of miR-15b-3p enhances tumorigenesis and malignant transformation through the DYNLT1/Caspase-3/Caspase-9 signaling pathway in gastric cancer
Source: J Exp Clin Cancer Res. 2020 Feb 10;39:32. doi: 10.1186/s13046-019-1511-6 (PMC7011526; doi:10.1186/s13046-019-1511-6)
Supplement: Supplementary file 7 — Additional file 7: Table S1. Real-time polymerase chain reaction primers. Table S2. Sequences of miR-15b-3p oligo. [file 13046_2019_1511_MOESM7_ESM.docx]

| **Table S1. Real-time polymerase chain reaction primers** | |
| --- | --- |
| **Gene** | **Sequences** |
| U6-F | 5'-CTCGCTTCGGCAGCACA-3' |
| U6-R | 5'-AACGCTTCACGAATTTGCGT-3' |
| GAPDH-F | 5'-GCGAGATCCCTCCAAAATCAA-3' |
| GAPDH-R | 5'-GTTCACACCCATGACGAACAT-3' |
| cel-miR-39-3p-F | 5'-ACACTCCAGCTGGG TCACCGGGTGTAAATC-3' |
| cel-miR-39-3p-R | 5'-CTCAACTGGTGTCGTGGAGTCGGCAATTCAGTTGAGCAAGCTGA-3' |
| hsa-miR-15b-3p-F | 5'-ACACTCCAGCTGGG CGAATCATTATTTGCT-3' |
| hsa-miR-15b-3p-R | 5'-CTCAACTGGTGTCGTGGAGTCGGCAATTCAGTTGAGTAGAGCAG-3' |
| hsa-miR-192-5p-F | 5'-ACACTCCAGCTGGGCTGACCTATGAATTG-3' |
| hsa-miR-192-5p-R | 5'-CTCAACTGGTGTCGTGGAGTCGGCAATTCAGTTGAGGGCTGTCA-3' |
| hsa-miR-21-5p-F | 5'-ACACTCCAGCTGGGTAGCTTATCAGACTGA-3' |
| hsa-miR-21-5p-R | 5'-CTCAACTGGTGTCGTGGAGTCGGCAATTCAGTTGAGTCAACATC-3' |
| hsa-miR-141-3p-F | 5'-ACACTCCAGCTGGGTAACACTGTCTGGTAA-3' |
| hsa-miR-141-3p-R | 5'-CTCAACTGGTGTCGTGGAGTCGGCAATTCAGTTGAGCCATCTTT-3' |
| hsa-miR-15b-5p-F | 5'-ACACTCCAGCTGGGTAGCAGCACATCATGG-3' |
| hsa-miR-15b-5p-R | 5'-CTCAACTGGTGTCGTGGAGTCGGCAATTCAGTTGAGTGTAAACC-3' |
| hsa-miR-185-5p-F | 5'-ACACTCCAGCTGGGTGGAGAGAAAGGCAGT-3' |
| hsa-miR-185-5p-R | 5'-CTCAACTGGTGTCGTGGAGTCGGCAATTCAGTTGAGTCAGGAAC-3' |
| hsa-miR-532-5p-F | 5'-ACACTCCAGCTGGGCATGCCTTGAGTGTAG-3' |
| hsa-miR-532-5p-R | 5'-CTCAACTGGTGTCGTGGAGTCGGCAATTCAGTTGAGACGGTCCT-3' |
| hsa-miR-331-3p-F | 5'-ACACTCCAGCTGGGGCCCCTGGGCCTATC-3' |
| hsa-miR-331-3p-R | 5'-CTCAACTGGTGTCGTGGAGTCGGCAATTCAGTTGAGTTCTAGGA-3' |
| hsa-miR-106b-3p-F | 5'-ACACTCCAGCTGGGCCGCACTGTGGGTACT-3' |
| hsa-miR-106b-3p-R | 5'-CTCAACTGGTGTCGTGGAGTCGGCAATTCAGTTGAG GCAGCAAG -3' |
| hsa-miR-17-5p-F | 5'-ACACTCCAGCTGGGCAAAGTGCTTACAGTGC-3' |
| hsa-miR-17-5p-R | 5'-CTCAACTGGTGTCGTGGAGTCGGCAATTCAGTTGAGCTACCTGC-3' |
| hsa-miR-30a-3p-F | 5'-ACACTCCAGCTGGGCTTTCAGTCGGATGTT-3' |
| hsa-miR-20a-3p-R | 5'-CTCAACTGGTGTCGTGGAGTCGGCAATTCAGTTGAGGCTGCAAA-3' |
| hsa-miR-501-3p-F | 5'-ACACTCCAGCTGGGAATGCACCCGGGCAAG-3' |
| hsa-miR-501-3p-R | 5'-CTCAACTGGTGTCGTGGAGTCGGCAATTCAGTTGAGAGAATCCT-3' |
| hsa-miR-200c-3p-F | 5'-ACACTCCAGCTGGGTAATACTGCCGGGTAAT-3' |
| hsa-miR-200c-3p-R | 5'-CTCAACTGGTGTCGTGGAGTCGGCAATTCAGTTGAGTCCATCAT-3' |

| **Table S2. Sequences of miR-15b-3p oligo** | |
| --- | --- |
| **Gene** | **Sequences** |
| hsa-miR-15b-3p mimics | 5'-CGAAUCAUUAUUUGCUGCUCUA-3' |
|  | 5'-GAGCAGCAAAUAAUGAUUCGUU-3' |
| hsa-miR-15b-3p inhibitor | 5'-UAGAGCAGCAAAUAAUGAUUCG-3' |
